# Selective Double Addition Reaction of an E‒H Bond (E = Si, B) to a C≡N Triple Bond of Organonitriles

**DOI:** 10.3390/molecules23112769

**Published:** 2018-10-25

**Authors:** Masumi Itazaki, Hiroshi Nakazawa

**Affiliations:** Department of Chemistry, Graduate School of Science, Osaka City University, Sumiyoshi-ku, Osaka 558-8585, Japan

**Keywords:** hydrosilylation, hydroborylation, dihydroborylsilylation

## Abstract

The catalytic double hydrometalation such as hydrosilylation and hydroborylation of organonitriles has attracted considerable attention because the obtained products are widely used in organic synthesis and it is thought to be one of the effective methods for reduction of organonitriles. However, the examples of these reactions are quite limited to date. This paper summarizes the development of selective double hydrosilylation, double hydroborylation, and dihydroborylsilylation of organonitriles, including their reaction mechanisms and the role of the metal species in the catalytic cycle.

## 1. Introduction

**Scheme 1:**
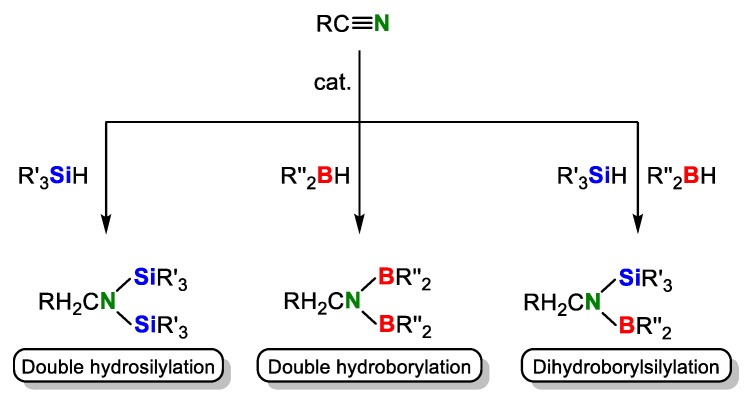
Double hydrosilylation, double hydroborylation, and dihydroborylsilylation of organonitriles.

**Figure 1:**
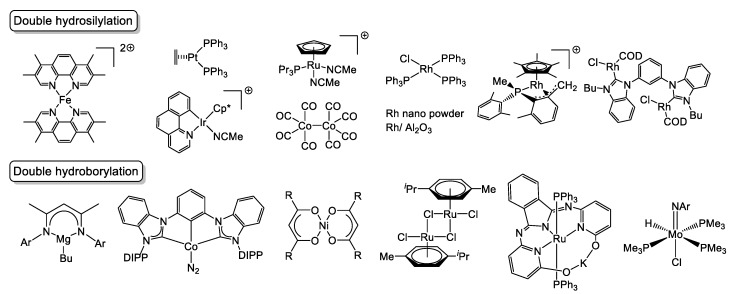
Metal catalysts for double hydrosilylation and double hydroborylation of organonitriles.

The catalytic hydrosilylation and hydroborylation of the carbon-nitrogen triple bond (C≡N bond) in organonitriles is becoming important in the synthetic chemistry. Although the term “hydroboration” is also widely used, we use “hydroborylation” in this paper from comparison with hydrosilylation. There is an advantage that these reactions do not generate by-products theoretically and the compounds with an N−Si or N−B bond thus obtained are useful products for the synthetic intermediates in organic chemistry. For examples, disilylamines (double hydrosilylation product) act as precursors for the production of Si,N-containing polymers [1,2,3,4], amine ligands for organometallic compounds [5,6], and silylating [7] and coupling [8] agents. Borylamines (hydroborylation products) have been reported to show a unique reactivity as iminium ion generators [9]. In addition, it is known that hydrosilylation and hydroborylation of a C≡N bond are one of effective methods to reduce organonitriles [10,11,12,13]. However, these hydrometalations do not occur under typical reaction conditions for hydrosilylation [14] and hydroborylation [15] because of the strong C≡N bond dissociation energy (179.3 kcal/mol, 750.0 kJ/mol) [16]. Actually, examples of catalytic double hydrometalation of the carbon-nitrogen triple bond (C≡N bond) in organonitriles are limited: one example of Fe [12], Pt [17], Ir [18], and Ru [19], two examples of Co [20,21], four examples of Rh [22,23,24,25], and main group elements and fluoride [13,26,27,28] for double hydrosilylation and one example of Mg [29], Co [30], and Ni [31], and two examples of Ru [32,33] and Mo [34,35] for double hydroborylation have been reported to date. Those metal catalysts are depicted in Figure 1. Although it is known that borylsilylamines are advantageous precursors for obtaining B/Si/N/C ceramics having a highly heat-resistant property [36,37], catalytic dihydroborylsilylation of organonitriles has not been achieved yet. (Scheme 1). In addition, the dual catalyst having both hydrosilylation and hydroborylation activities has not been found.

## 2. Double Hydrosilylation of Organonitriles

In 1982, Corriu and co-workers reported that the reaction of 1,4-dicyanobutane with 1,2-bis(dimethylsilyl)benzene in the presence of a catalytic amount of RhCl(PPh_3_)_3_ afforded a mixture of *trans-N,N*-disilylenamines (major product) and *N,N*-disilylamines (minor product) (Equation (1)) [23]. In the case of benzonitrile, only double hydrosilylation product was obtained, although the yield of the product was low (Equation (2)):
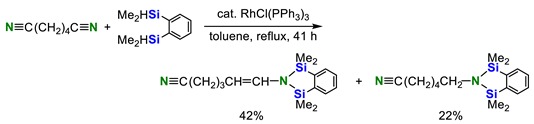
(1)


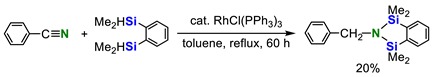
(2)

**Scheme 2:**
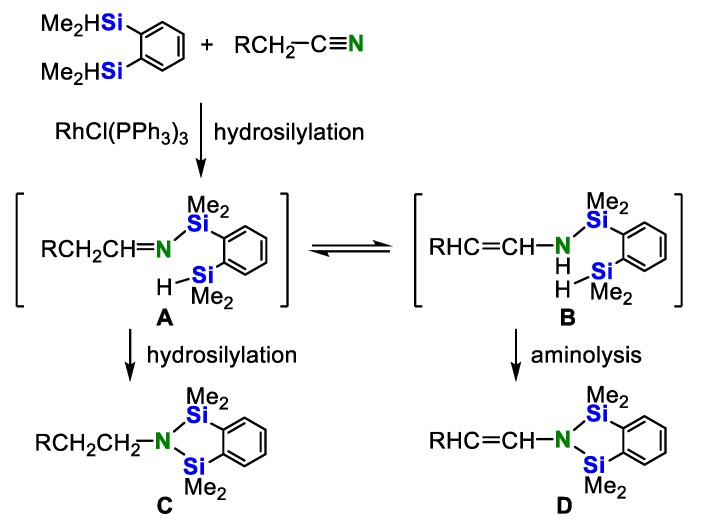
Proposed mechanism for double hydrosilylation of organonitriles with 1,2-bis(dimethylsilyl)benzene.

The proposed reaction mechanism of Rh-catalyzed double hydrosilylation of organonitriles with 1,2-bis(dimethylsilyl)benzene is shown in Scheme 2. Intermediates **A** and **B** are generated via the first hydrosilylation and they may be in equilibrium. The second hydrosilylation of **A** takes place to give the *N,N*-disilylamine (double hydrosilylation product) **C**. On the other hand, intermediate **B** is converted into the *N,N*-disilylenamine **D** as a result of aminolysis.

Murai’s group found the double hydrosilylation of aromatic and aliphatic nitriles catalyzed by a cobalt carbonyl Co_2_(CO)_8_ in 1985 and 1990 [20,21]. The desired products were obtained in the reaction of various aromatic nitriles with 10 equiv. of hydrosilane at 60 °C for 20 h in the presence of Co_2_(CO)_8_ (Table 1). The system possesses an excellent degree of functional group tolerance for the functionalized benzonitriles with electron-withdrawing or -donating groups such as Me, OMe, Cl, NMe_2_, CN, and CO_2_Me in the *para* position on the aryl ring. A Me group in *meta* position shows good reactivity, whereas that in the *ortho* position shows low reactivity.

Furthermore, aliphatic nitriles are adaptable to this reaction system and gave the corresponding products in moderate to excellent yields when PPh_3_ is added to the reaction system (Table 2). It is thought that a silylcobalt complex R_3_SiCo(CO)_4_, which is prepared by the reaction of Co_2_(CO)_8_ with R_3_SiH, is an important catalytic active spices in this system.

**Scheme 3:**
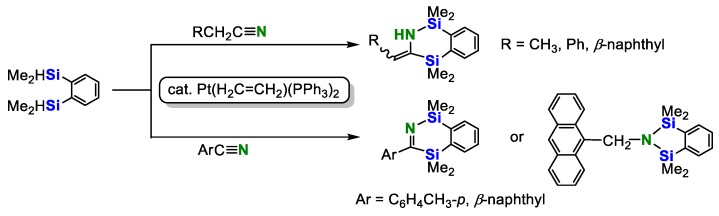
A platinum-catalyzed reaction of various nitriles with 1,2-bis(dimethylsilyl)benzene.

A platinum-catalyzed reaction of various nitriles with 1,2-bis(dimethylsilyl)benzene was reported by Tanaka’s group in 1992 (Scheme 3) [17]. In the presence of Pt(H_2_C=CH_2_)(PPh_3_)_2_ catalyst, reactions of aliphatic nitriles with 1,2-bis(dimethylsilyl)benzene gave the *N*-silyl enamies, while aryl nitriles were converted into the corresponding imines in high to excellent yields. The double hydrosilylation product was yielded in 64% when 9-anthroylnitrile was used.

In 1999, double hydrosilylation of arylnitriles catalyzed by heterogenous Rh powder and rhodium on γ-alumina was achieved by Pertici and co-workers (Table 3) [22]. The tendency of the reaction is similar to the Murai’s report [20]. The desired product was not obtained when the substrate with a Me group in the *ortho* position on the aryl ring was used. In addition, the yields decreased when HSi(OEt)_3_ as a hydrosilane or rhodium on γ-alumina instead of Rh powder was used.

Selective catalytic hydrosilylation of nitriles was found by Nikonov and Gutsulyak in 2010 [19]. The reaction of organonitriles with HSiMe_2_Ph in a 1:1 molar ratio afforded the corresponding imines. In addition, the *N,N*-disilylamines were produced by the reaction of organonitriles with 2.5 equiv. of HSiMe_2_Ph although a long reaction time was required (Table 4). In the case of isobutyronitrile, the mixture of *N,N*-disilyenamine (57%) and *N,N*- disilylamines (43%) were yielded.

**Scheme 4:**
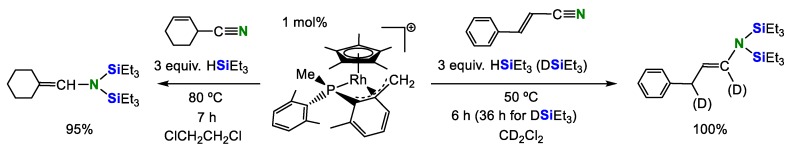
Rh-catalyzed double hydrosilylation of α,β-unsaturated nitriles to vinyl amines.

Rhodium-catalyzed hydrosilylation of α,β-unsaturated nitriles into vinylamines was achieved by Carmona’s group in 2011 (Scheme 4) [24]. Acetonitrile showed low activity (<40%) and benzonitrile did not undergo hydrosilylation in this catalytic system.

**Scheme 5:**
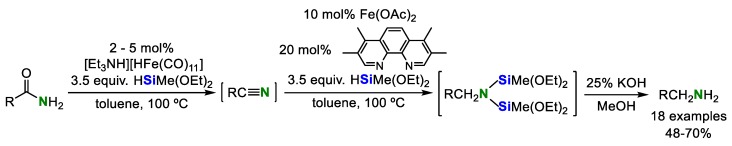
Reductions of aromatic and aliphatic primary amides to amines catalyzed by two iron complexes.

Beller’s group achieved the conversion of aromatic and aliphatic primary amides into amines catalyzed by two iron cooperative catalytic system in 2012 [12]. In this system, the combination of Fe(OAc)_2_ and phenanthroline ligand acts as a catalyst for the double hydrosilylation of aromatic and aliphatic nitriles, which are prepared by reduction of amides catalyzed by an iron complex [Et_3_NH][HFe_3_(CO)_11_] (Scheme 5).

**Scheme 6:**
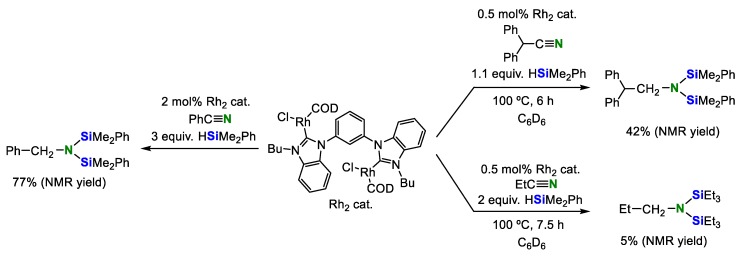
Homobimetallic Rh complex-catalyzed hydrosilylation of nitriles.

In 2013, Hollis and co-workers reported that a homobimetallic Rh complex having an NHC ligand also acted as a catalyst for the double hydrosilylation (Scheme 6) [25]. Benzonitrile was converted into the corresponding product in good yield. For aliphatic nitriles, the activity of diphenylacetonitrile was higher than that of propionitrile (the yields of the corresponding disilylamines were 42% and 5%, respectively).

In 2017, Djukic and co-workers reported an iridacycle complex as a catalyst for the conversion of organonitriles into *N,N*-disilylamines by the double hydrosilylation [18]. The reaction was adaptable to a wide variety of aromatic nitriles (Table 5). In this system, Cl and F groups in *ortho* position on the aryl ring did not disturb the double hydrosilylation, whereas a nitrile having a coordination-feasible substituent did the reaction.

**Scheme 7:**
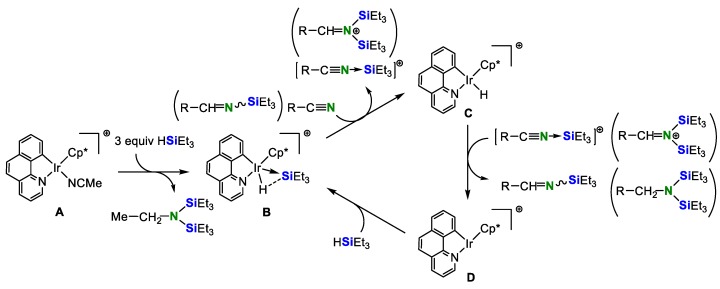
Plausible reaction pathway of the double hydrosilylation of nitriles catalyzed by a cationic iridium complex.

Scheme 7 depicts a plausible reaction pathway of the double hydrosilylation of nitriles catalyzed by a cationic iridium complex **A**. The reaction of **A** with 3 equiv. of HSiEt_3_ affords the silane−iridacycle adduct **B** and EtN(SiEt_3_)_2_ as a result of electrophilic and heterolytic activation of the Si−H bond. Subsequently, the abstraction of the SiEt_3_ group in **B** by nitrile gives the hydrido complex **C** and the *N*-silylnitrilium cation and then the hydrido transfer from **C** to the *N*-silylnitrilium cation produces the *N*-silylimine and unsaturated Ir complex **D**. Finally, **D** reacts with HSiEt_3_ to regenerate **B**. A similar reaction proceeds once again to give the desired *N,N*-disilylamine.

**Scheme 8:**
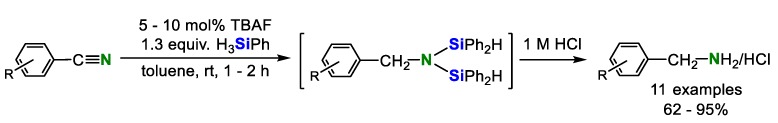
TBAF catalyzed hydrosilylation for the reduction of aromatic nitriles.

Recently, some metal-free hydrosilylation reactions of organonitriles were achieved. Beller’s group reported TBAF catalyzed hydrosilylation for the reduction of aromatic nitriles in 2013 (Scheme 8) [13]. Various aryl nitriles were converted into the corresponding benzylamines via *N,N*-disilylamines. Heterocyclic nitriles such as 3-thiophenecarbonitrile and picolinonitrile, as well as hexanenitrile showed no activity.

**Scheme 9:**
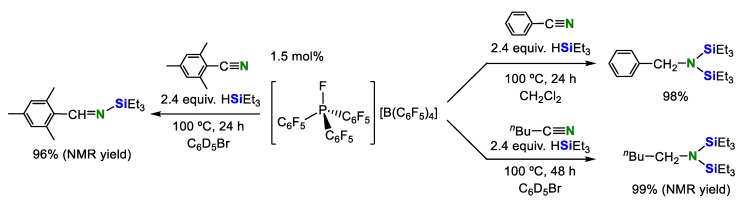
[(C_6_F_5_)_3_PF][B(C_6_F_5_)_4_]-catalyzed hydrosilylation of nitriles.

In 2015, Grimme, Stephan and co-workers found that an electrophilic phosphonium salt, [(C_6_F_5_)_3_PF][B(C_6_F_5_)_4_] acted as a catalyst for the double hydrosilylation of organonitriles. In this system, benzonitrile and propionitrile were converted into the corresponding *N,N*-disilylamines in quantitative yields. The *N*-silylimine was selectively formed in excellent yield when sterically bulky mesityl nitrile was used (Scheme 9) [26].

**Scheme 10:**
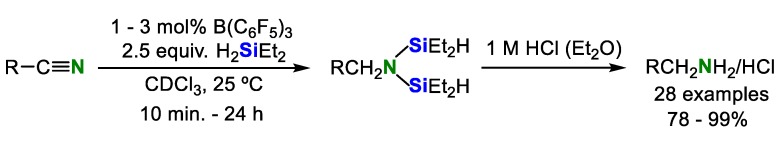
[B(C_6_F_5_)_3_]-catalyzed double hydrosilylation of aryl and alkyl nitriles.

In the same year, Chang’s group reported that tris(pentafluorophenyl)borane [B(C_6_F_5_)_3_]-catalyzed silylative reduction of conjugated nitriles to β-silyl amines as a result of selective double hydrosilylation of the C≡N bond and hydrosilylation of the C=C bond [27]. Table 6 summarizes the scope and limitation of substrates for this reaction. The system possesses a good degree of functional group tolerance for the functionalized conjugated nitrile with an electron-withdrawing or -donating group on the aryl ring. Furthermore, this catalytic system was also applicable to aryl and alkyl nitriles (Scheme 10) [28].

In the cases of the catalytic double hydrosilylation, an excess amount of the hydrosilane over the organonitrile was required for the selective formation of the desired disilylamines. Therefore, a new synthetic strategy without using an excess amount of the hydrosilane and a new catalyst have been demanded.

In order to create a new approach to double hydrosilylation of organonitriles, we focused on transition metal complexes with Z-type ligand(s). The interaction of a Z-type ligand (acts as a two-electron acceptor, a Lewis acid) with a late transition metal (acts as a two-electron donor, a Lewis base) has attracted considerable attention as a new approach for controlling the electronic characteristics and reactivity of the metal center [38,39,40,41,42,43,44,45,46,47]. This approach is becoming increasingly important in the field of catalysts [48,49,50,51,52,53,54,55,56]. Previously, we reported that triruthenium dodecacarbonyl Ru_3_(CO)_12_ reacted with InX_3_ (X = Cl, Br) to yield the first ruthenium(0) indane complex, *fac-*[Ru(NCCH_3_)_3_(CO)_2_(InX_3_)] (X = Cl (**1Cl**), Br (**1Br**)). In addition, the reaction of **1Cl** and **1Br** with 1 equiv. of PPh_3_ afforded *cis,cis,trans*-[Ru(NCCH_3_)_2_(CO)_2_(InX_3_)(PPh_3_)] (X = Cl (**2Cl**), Br (**2Br**)) as a result of selective replacement of CH_3_CN(*trans* to InX_3_) by PPh_3_ (Equation (3)) [57]. It is considered that the InX_3_ in **1Cl**, **1Br**, **2Cl**, and **2Br** is a Z-type ligand.


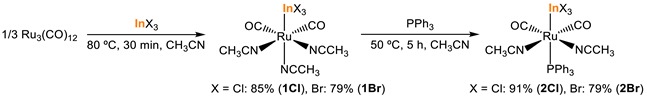
(3)

On the other hand, the reaction of triiron dodecacarbonyl Fe_3_(CO)_12_ with InX_3_ afforded the iron complex containing indium ligands [Fe(NCCH_3_)_6_][*cis-*Fe(CO)_4_(InX_3_)_2_] (X = Cl (**3Cl**), Br (**3Br**), I (**3I**)) (Equation (4)) [58]. These complexes represent the first example of transition metal complexes containing two terminal indium fragments. For the anionic iron complex [*cis*-Fe(CO)_4_(InCl_3_)]^2–^, the ^57^Fe Mössbauer and IR data suggest that the Fe^0^(CO)_4_ has two [Fe–In–X_3_]^−^ portions like [InX_4_]^−^ called as “indate”.


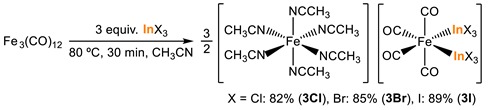
(4)

As there had been no organic reactions catalyzed by a combination of an iron complex and an indium source, the catalytic ability of the iron complex was examined for double hydrosilylation of organonitriles, and it was found interesting knowledge. The results were described in a bit more detail below.

The reaction of CH_3_CN with HSiMe_2_Ph in the presence of a catalytic amount of **3Cl** produced CH_3_CH_2_N(SiMe_2_Ph)_2_ in 85% yield (Equation (5)) [59]. It should be noted that this catalytic reaction provided the double hydrosilylation product selectively in spite of using an excess amount of acetonitrile over the hydrosilane.


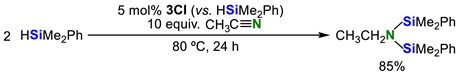
(5)

Complex **3Cl** was a better catalyst than **3B** and **3I**. The catalytic activity of a mixture of dodecacarbonyltriiron Fe_3_(CO)_12_ and indium trichloride (InCl_3_) was similar to that of **3Cl**, whereas the double hydrosilylation did not proceed when either Fe_3_(CO)_12_ or InCl_3_ was used. It was revealed that not the cationic iron complex [Fe(NCCH_3_)_6_]^2+^ but the anionic iron complex [*cis*-Fe(CO)_4_(InCl_3_)]^2-^ of **3Cl** played a crucial role in the double hydrosilylation. Various aliphatic and aromatic nitriles (RC≡N, for which R = Me, Et, *^i^*Pr, *^i^*Bu, Ph, *p*-Tol, *m*-Tol or *o*-Tol) underwent the double hydrosilylation without the formation of the single hydrosilylation compound (Table 7). In the double hydrosilylation of propanenitrile (EtCN), the expected product EtCH_2_N(SiMe_2_Ph)_2_ was obtained as the main product along with a little amount of MeCH_2_N(SiMe_2_Ph)_2_ (2% yield as determined by NMR). The latter product is considered to be derived from the dissociated CH_3_CN obtained by the MeCN/EtCN ligand exchange on the iron center of the cationic part in **3Cl**. The yields of the products decreased when going from *p*- to *m*- and *o*-tolunitrile (55, 49, and 41%, respectively), presumably due to steric effects. This catalytic system was also applicable to 4-pyridinecarbonitrile although the yield of the corresponding disilylamine was low (21%). No reaction occurred for *^t^*BuCN, CCl_3_CN, and C_6_F_5_CN with HSiMe_2_Ph. These results indicate that organonitriles having a bulky or an electro-withdrawing group are unfavorable for the double hydrosilylation. The double hydrosilylation reaction of MeCN did not proceed when a bulkier hydrosilane (HSiMePh_2_) was used. Instead, the reactions of HSiMe_2_Fc with *p*-TolCN and H_2_SiMePh with MeCN provided the double hydrosilylation compounds in 43% and 76% yields, respectively.

## 3. Double Hydroborylation of Organonitriles

**Scheme 11:**
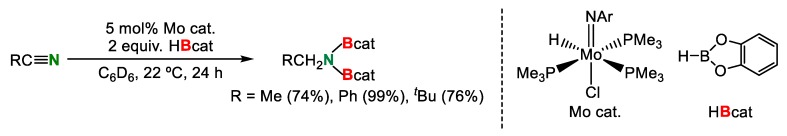
Double hydroborylation of nitriles catalyzed by an imido-hydrido Mo(IV) complex.

Nikonov and co-worker found that the imido-hydrido Mo(IV) complex acted as a catalyst for the double hydroborylation of organonitriles in 2012 [34] and 2015 [35]. The reaction of organonitriles RCN (R = Me, Ph, *^t^*Bu) with 2 equiv. of HBcat (catecholborane) in the presence of a catalytic amount of imido-hydrido Mo(IV) complex afforded the corresponding *N,N*-diborylamines in good to excellent yields (Scheme 11).

**Scheme 12:**
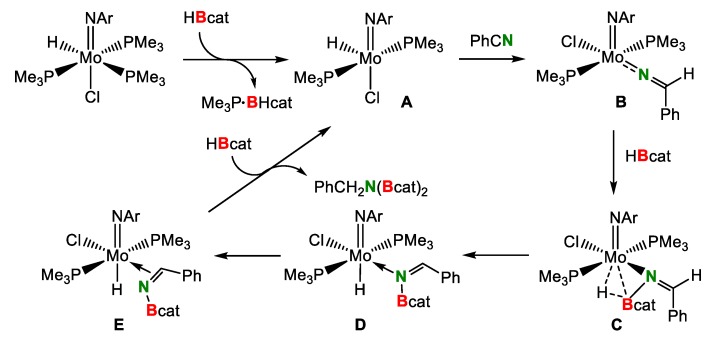
Plausible reaction pathway of the double hydroborylation of nitriles catalyzed by an imido-hydrido Mo(IV) complex.

A plausible reaction pathway of the double hydroborylation of nitriles catalyzed by the imido-hydrido Mo(IV) complex was shown in Scheme 12. The abstraction of the coordinated PMe_3_ ligand by HBcat results in the formation of unsaturated Mo complex A. Subsequently, the reaction of A with PhCN affords benzylideneamide complex B and then B reacts with HBcat to yield agostic amido-borane adduct complex C. Complex C is converted into borylimine complex E through *N*-coordinated borylimine complex D. Finally, the elimination of the desired *N,N*-diborylamine from the Mo center in E regenerates catalytic intermediate A to complete the catalytic cycle.

**Scheme 13:**
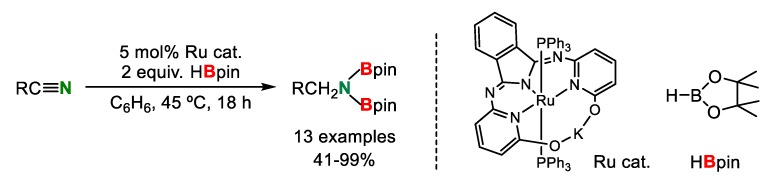
Ru-catalyzed double hydroborylation of nitriles.

In 2015, Szymczak’s group reported that catalytic nitrile hydroborylation using a ruthenium complex having a bifunctional pincer ligand took place for several *p*-substituted aryl nitriles with HBpin (pinacolborane) to give the corresponding diborylamines in moderate to excellent yields (Scheme 13) [33].

**Scheme 14:**
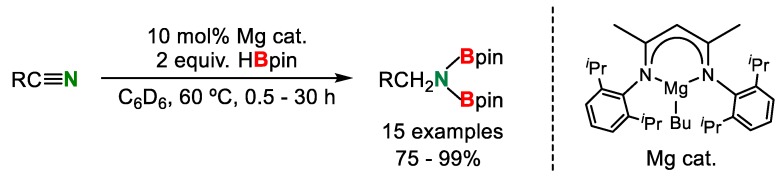
Double hydroborylation of nitriles catalyzed by a Mg complex.

The double hydroborylation of organonitriles by HBpin was also reported by Hill’s group in 2016. In this reaction, a β-diketiminato *n*-butylmagnesium complex was found to be an efficient catalyst and the desired products were obtained in good to excellent yields (Scheme 14) [29]. This reaction showed good functional group tolerance. In addition, benzonitrile having a Me group in the *ortho* position on the aryl ring also showed good reactivity (86%).

**Scheme 15:**
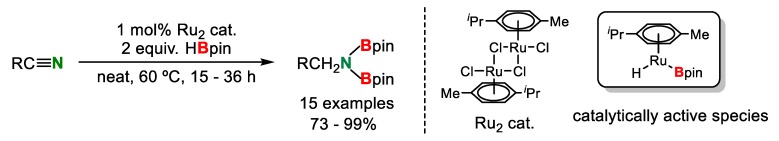
Homobimetallic Ru-catalyzed hydroborylation of nitriles.

At almost the same time, Gunanathan’s group reported the selective conversion of nitriles into amines by double hydroborylayion [32]. Various organonitriles reacted with 2 equiv. of HBpin in the presence of a catalytic amount of homobimetallic Ru complex [Ru(*p*-cymene)Cl_2_]_2_ to obtain the correspsonding *N,N*-diborylamines in good to excellent yields (Scheme 15). It was thought that a boryl hydrido complex [Ru(*p*-cymene)H(Bpin)], which was prepared by the reaction of [Ru(*p*-cymene)Cl_2_]_2_ with HBpin, was an important catalytically active species in this system.

In 2017, Fout’s group reported the double hydroborylation of organonitriles catalyzed by a Co(I) complex [30]. In this system, alkyl and (hetero)aryl nitriles were converted into the desired *N,N*-diborylamines in moderate to high yields (Table 8).

**Scheme 16:**
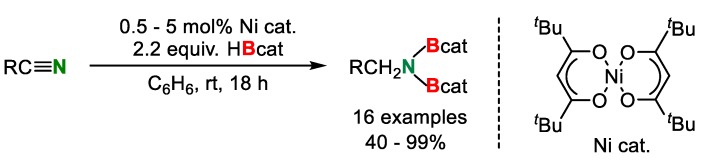
Ni complex-catalyzed hydroborylation of nitriles.

In the same year, a nickel catalyzed double hydroborylation of organonitriles was achieved by Nakajima, Shimada and co-workers [31]. The reaction of organonitriles with 2.2 equiv. of HBcat yielded *N,N*-diborylamines in moderate to excellent yields (Scheme 16). The reaction was applicable to a wide variety of nitriles whereas benzonitrile having a Me group in the *ortho* position on aryl ring (40%) and 2-thienyl nitrile (47%) showed lower reactivities.

**Scheme 17:**
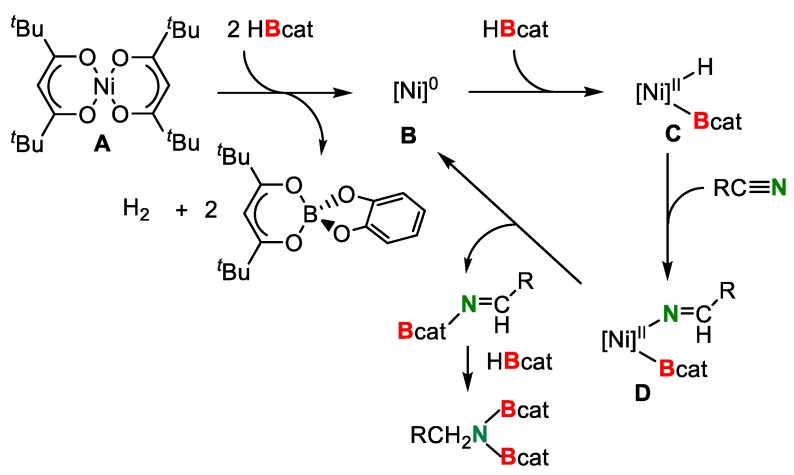
Proposed mechanism for Ni-catalyzed double hydroborylation of nitriles.

A proposed mechanism is shown in Scheme 17. The reduction of A by 2 equiv. of HBcat produces the active Ni(0) species B. Oxidative addition of H–Bcat toward the Ni(0) center gives boryl hydrido intermediate C. Insertion of a nitrile into the Ni–H bond in C affords D. The subsequent reductive elimination of the borylimine from D regenerates an intermediate B to complete the catalytic cycle. The obtained borylimine further reacts with HBcat to give the *N,N*-diborylamine.

We also reported the catalytic activity of **3Cl** for the double hydroborylation of organonitriles (Table 9) [60]. The tendency of the double hydroborylation by **3Cl** was similar to that of the double hydrosilylation. In the double hydroborylation, *^t^*BuCN was also converted into the corresponding product in good yield. No reaction occurred for CCl_3_CN, C_6_F_5_CN, and 4-PyCN with HBpin, suggesting that a strong electron-withdrawing substituent, or a coordination-feasible substituent on the nitrile carbon retards or disturbs the double hydroborylation of the nitrile portion. The molecular structures of EtN(Bpin)_2_ and PhCH_2_N(Bpin)_2_ were confirmed by single-crystal X-ray structure diffraction analyses. These structures showed the formation of diborylamine as results of the selective double hydroborylation of organonitriles.

## 4. Dihydroborylsilylation of Acetonitrile

With the hope of selective formation of borylsilylamine in the Fe-In cooperative catalytic system, the reaction of acetonitrile with both hydrosilane and hydroborane was investigated [60]. The mixture of acetonitrile (4.0 mmol), HSiMe_2_Ph (0.4 mmol), HBpin (0.4 mmol), and **3Cl** (0.04 mmol) was stirred at 80 °C for 24 h under an argon atmosphere (Equation (6)). The desired borylsilylamine EtN(SiMe_2_Ph)(Bpin) was obtained with high selectivity although diborylamine was also generated in 6% NMR yield. The isolation of EtN(SiMe_2_Ph)(Bpin) in 81% yield was achieved by the distillation using a Kugelrohr in a glove box. This reaction is the first one-pot synthesis of borylsilylamine via catalytic hydrosilylation and hydroborylation.


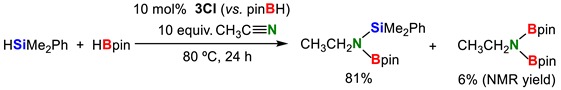
(6)

In order to obtain insight into the reaction pathway of our catalytic system, we checked the double hydrosilylation under the similar reaction conditions in Entry 1 in Table 1 in the presence of 5 equiv. of InCl_3_ (Equation (7)). The expected double hydrosilylation product was not obtained. Therefore, we thought that the dissociation of InCl_3_ from the iron center in **3Cl** was one of the key steps in our system.


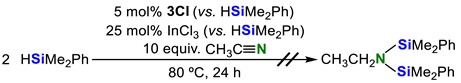
(7)

Baba and co-worker reported that the indium trihalide InX_3_ reacted with hydrosilane to give indium hydride HInX_2_ and this compound acted as a radical [61]. If the elimination of InCl_3_ occurs from the iron center in **3Cl**, the released InCl_3_ seems to react with hydrosilane to yield the corresponding indium hydride HInCl_2_. Therefore, we examined our reaction system in the presence of TEMPO (2,2,6,6-tetramethylpiperidine-1-oxyl) as a radical scavenger and found that the trace amount of the disilylamine was yielded (Equation (8)). This result showed that HInCl_2_ was involved in the reaction pathway:
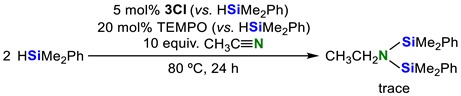
(8)

When deuterated acetonitrile (CD_3_CN) was used in place of CH_3_CN under the same reaction conditions in Entry 1 in Table 1, CD_3_CH_2_N(SiMe_2_Ph)_2_ was obtained in 73% yield (Equation (9)):
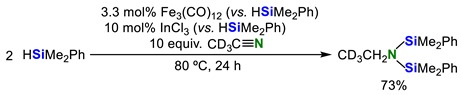
(9)

**Scheme 18:**
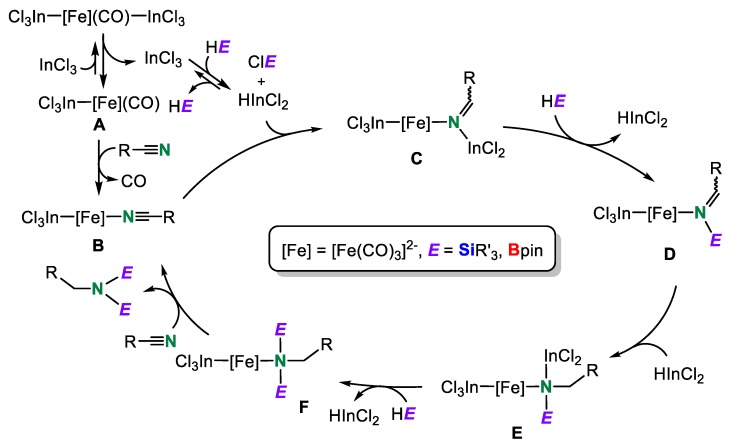
Proposed catalytic cycle for double hydrosilylation, double hydroborylation, and dihydroborylsilylation of organonitriles in the presence of **3Cl**.

Based on the results mentioned above, we proposed a tentative catalytic cycle for the double hydrosilylation, double hydroborylation, and dihydroborylsilylation of organonitriles in the presence of **3Cl** (Scheme 18). Dissociation of one of the coordinated InCl_3_ ligands from [Fe(InCl_3_)_2_(CO)_4_]^2–^ occurs to give free InCl_3_ and monoindium-iron complex [Fe(InCl_3_)(CO)_4_]^2–^ **A**. Then, the reaction of the eliminated InCl_3_ with H*E* (*E* = SiR’_3_, Bpin) provides HInCl_2_ and Cl*E*. The formation of ClBpin was confirmed by the NMR measurement of the reaction mixture of HBpin and InCl_3_ in acetnitrile-*d*_3_. On the other hand, release of one carbonyl ligand from **A** and successive coordination of nitrile takes place to form nitrile complex [Fe(InCl_3_)(CO)_3_(NCR)]^2–^ **B**. Complex **B** reacts with HInCl_2_ to generate indylimine iron intermediate **C**, followed by the reaction with H*E* to yield imine iron complex **D** and HInCl_2_. A similar reaction proceeds once again to give indane amine iron complex **F** through **E**. Finally, the elimination of the corresponding amine compound from the iron center in **F** and then recoordination of an organonitrile to the iron center yields catalytic intermediate **B** to complete the catalytic cycle. We believe that the imine moiety in **D** may not dissociate, causing selective formation of the corresponding amine compounds in this catalytic system.

## 5. Conclusions

There is growing interest in the selective double addition reaction of an E‒H bond (E = Si, B) to a C≡N triple bond of organonitriles because two N‒Si bonds or two N‒B bonds can be generated in one pot. Great efforts to establish catalytic system of such double addition by many research groups have resulted in several outstanding findings to date. Although some reaction mechanisms have been proposed, there are many unclear points from a mechanistic point of view.

We also have been engaged in creation of new catalytic systems for double hydrosilylation and double hydroborylation of organonitriles, and found a new catalytic system in which both iron and indium serve cooperatively. In addition, we found that this catalytic system could be applicable to the first single-step synthesis of borylsilylamine. The consideration of the reaction mechanism suggested that the anionic iron complex [*cis*-Fe(CO)_4_(InCl_3_)]^2-^ was an important catalytic precursor.

Selective double addition of an E‒H bond to a C≡C triple bond and a C≡E triple bond (not only a C≡N triple bond but also other C≡heteroatom triple bonds) is becoming promising. More investigation concerning creation of new catalytic systems and elucidation of reaction mechanisms are expected to be continued.

## Figures and Tables

**Table 1:**
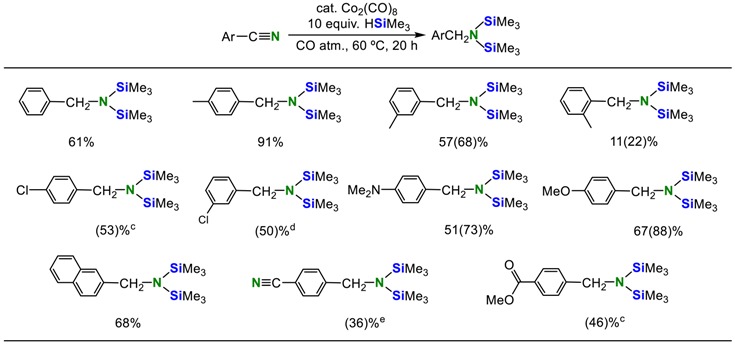
Cobalt carbonyl catalyzed double hydrosilylation of aromatic nitriles ^a,b^.

^a^ Reaction conditions: nitrile (2.5 mmol), HSiMe_3_ (25 mmol), Co_2_(CO)_8_ (0.2 mmol), toluene (10 mL). ^b^ GLC yields in parentheses. ^c^ 40 h. ^d^ 48 h. ^e^ Co_2_(CO)_8_ (0.625 mmol) was used.

**Table 2:**
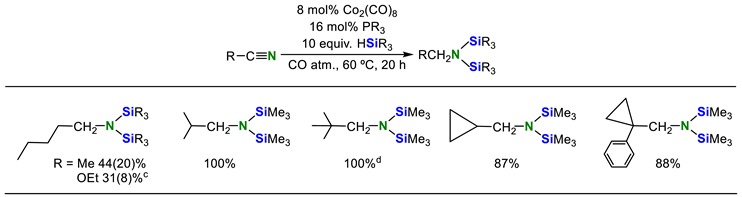
Cobalt carbonyl catalyzed double hydrosilylation of aliphatic nitriles ^a,b^.

^a^ Reaction conditions: nitrile (2.5 mmol), HSiMe_3_ (25 mmol), Co_2_(CO)_8_ (0.2 mmol), PPh_3_ (0.4 mmol), toluene (10 mL). ^b^ The yields of enamine in parentheses. ^c^ HSi(OEt)_3_ (10 mmol) and toluene (5 mL) were used. ^d^ P(OEt)_3_ (0.4 mmol) was used.

**Table 3:**
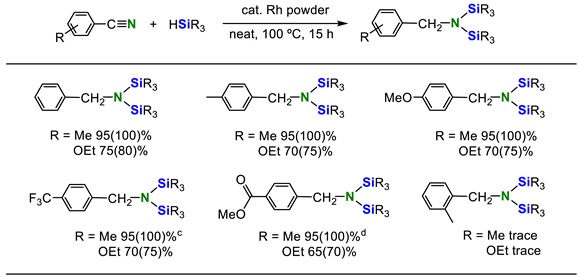
Double hydrosilylation of aromatic nitriles promoted by rhodium metal particles ^a,b^.

^a^ Reaction conditions: nitrile (9.8 mmol), hydrosilane (49 mmol), rhodium (0.1 mg atom). ^b^ GLC conversion of the nitriles in parentheses. ^c^ 4 h. ^d^ 7 h.

**Table 4:**
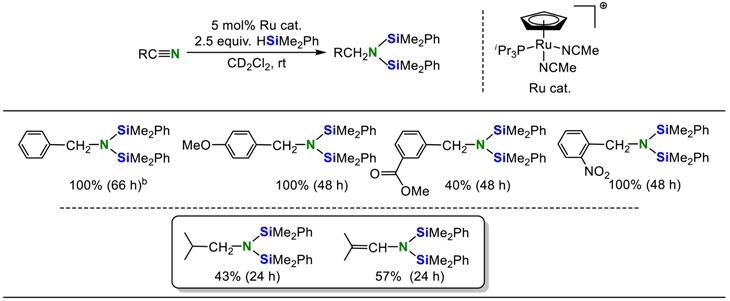
Ru-catalyzed double hydrosilylation of nitriles ^a^.

^a^ Reaction conditions: nitrile (0.13 mmol), HSiMe_2_Ph (0.33 mmol), Ru cat. (7.0 mg). ^b^ Nitrile (0.08 mmol), HSiMe_2_Ph (0.19 mmol), Ru cat. (3.0 mg), and CD_3_Cl instead of CD_2_Cl_2_ were used.

**Table 5:**
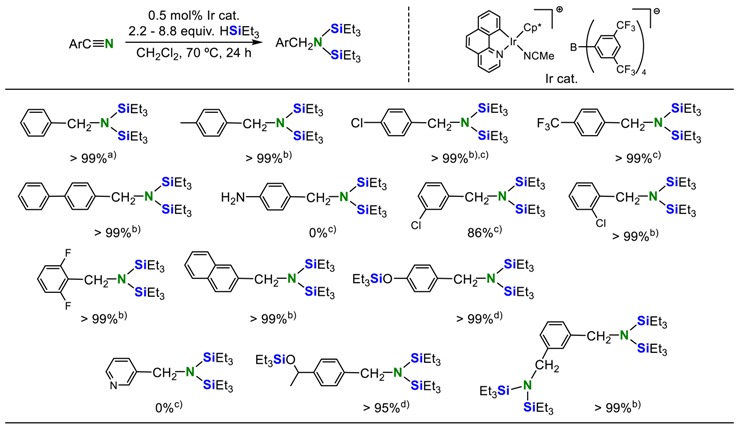
Ir-catalyzed double hydrosilylation of aryl nitriles ^a^.

^a^ Conditions: (a) nitrile (1.1 mmol), HSiEt_3_ (0.35 mL, 2.1 mmol), Ir cat. (6.8 mg, 4.8 mmol [0.5 mol %] or 13.7 mg, 9.7 mmol [1 mol %]), 70 °C; (b) aryl nitrile (0.8 mmol), HSiEt_3_ (0.3 mL, 1.8 mmol), Ir cat. ([0.5 mol %] or [1 mol %]), 70 °C; (c) conditions similar to those in (b), except that instead of 2.2 equiv. of HSiEt_3_, 4.4 equiv. (0.60 mL, 3.7 mmol) was used; (d) conditions similar to those in (b), except that instead of 2.2 equiv. of HSiEt_3_, 8.8 equiv. (1.20 mL, 7.5 mmol) was used. Yields were determined by ^1^H NMR spectroscopy using 1,3,5-tri-tert-butylbenzene as internal reference.

**Table 6:**
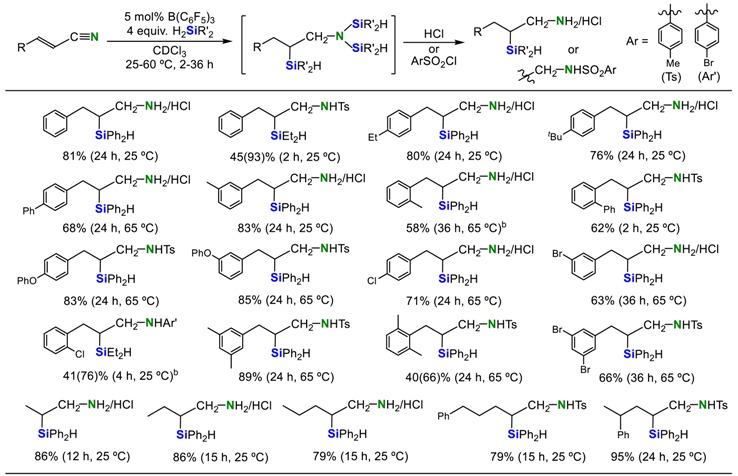
[B(C_6_F_5_)_3_]-catalyzed hydrosilylation of conjugated nitriles ^a^.

^a^ Conditions: Substrate (0.5 mmol), silane (4 equiv), and B(C_6_F_5_)_3_ (5 mol%). Yield of isolated product given and value within parentheses is the yield of the initially formed β-silyl-*N,N*-disilylamine using 1,1,2,2-tetrachloroethane as an internal standard. ^b^ B(C_6_F_5_)_3_ (7 mol%).

**Table 7:**
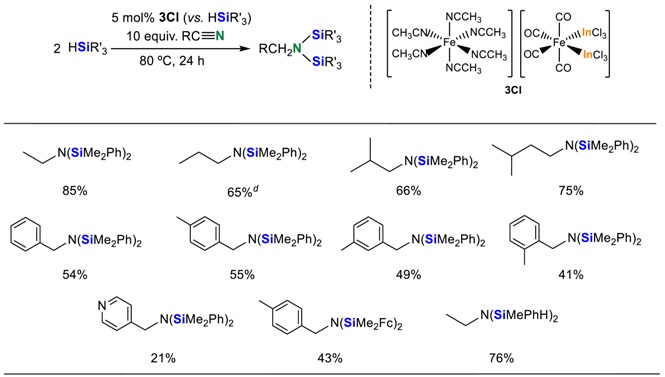
Double hydrosilylation of organonitriles in the presence of **3Cl** ^a,b,c^.

^a^ Reaction conditions: organonitrile (4.0 mmol), hydrosilane (0.80 mmol), **3Cl** (0.040 mmol); ^b^ Isolated yield; ^c^ Fc stands for a ferrocenyl group; ^d^ Little amounts of by-product EtN(SiMe_2_Ph)_2_ were removed by distillation.

**Table 8:**
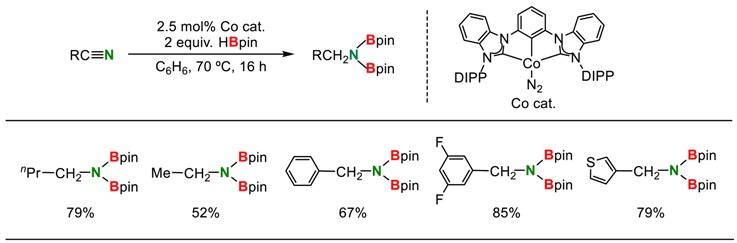
Double hydroborylation of organonitriles in the presence of a Co complex.

**Table 9:**
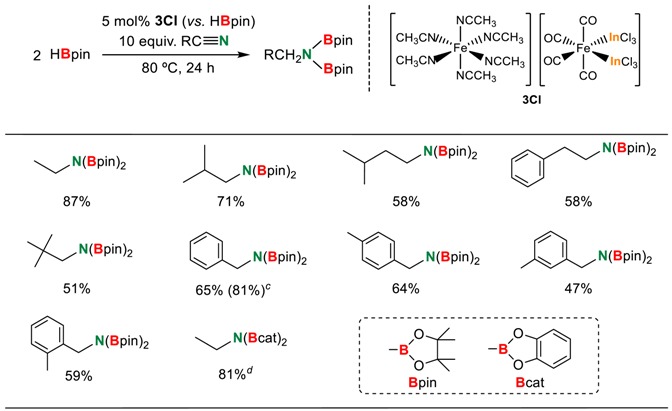
Double hydroborylation of organonitriles in the presence of **3Cl** ^a,b^.

^a^ Reaction conditions: organonitrile (4.0 mmol), HBpin (0.80 mmol), **3Cl** (0.040 mmol); ^b^ Isolated yield; ^c^ 10 mol% **3Cl** was used; ^d^ Catechol borane was used at room temperature for 18 h.
